# Serum Uric Acid Levels and Risk of Atrial Fibrillation in Heart Failure Patients

**DOI:** 10.2174/011573403X379855250620115105

**Published:** 2025-06-27

**Authors:** Mounika Reddy Vadiyala, Jeffy Varghese, Kesar Prajapati, Qiming Dong, Rupak Desai

**Affiliations:** 1 Department of Internal Medicine, Maimonides Medical Center, Brooklyn, NY 11219, USA;; 2 Department of Internal Medicine, Metropolitan Hospital Center, New York, NY 10029, USA;; 3 Department of Internal Medicine, Greater Baltimore Medical Center, Towson, MD 21204, USA;; 4 Independent Outcomes Researcher, Atlanta, GA, USA

**Keywords:** Cardiovascular diseases, uric acid, hyperuricemia, heart failure, atrial fibrillation, SUA levels

**TO THE EDITOR,**

Heart failure (HF) and atrial fibrillation (AF) are major contributors to cardiovascular diseases, often co-existing and leading to the development or progression of the other. Hyperuricemia, defined as a serum uric acid level (SUA) >7, and atrial fibrillation (AF) have been independently associated with increased morbidity and mortality in patients with HF [1, 2]. Emerging evidence further associates increased SUA levels with AF. These patients were noted to have increased left ventricular (LV) and left atrial (LA) remodeling from damage due to oxidative stress [3]. However, there continues to be a paucity of evidence exploring the connection between SUA levels and the risk of AF in patients with HF. This letter presents a brief critical review on three published studies on the influence of SUA levels on the likelihood of developing AF in individuals with HF. The review included data from 17,582 patients across the three studies. The findings from each study are summarized in this letter.

The research conducted by Tekin *et al.* (2012) focused on a group of 363 individuals with heart failure (HF) divided into two categories: 78 patients with fibrillation (AF) and 285 patients with normal heart rhythms [4]. The selection criteria were strict, excluding individuals who had recently suffered a heart attack, severe kidney problems, infections, or active cancer. The research aimed to investigate how serum acid (SUA) levels are linked to AF in the study group. They discovered that individuals in the AF category had higher SUA levels (6.4 ± 2.09 mg/dL) than those in the non-AF category (5.7 ± 1.9 mg/dL), with an odd’s ratio of 1.27 (1.06±1.52, *p*-value 0.01). Through logistic regression analysis, they identified age, SUA, left atrial diameter, left ventricular end-diastolic diameter, and left ventricular end-diastolic volume as key and independent predictors of AF occurrence.

Zhao *et al.*, in a study (2012), encompassed a cohort of 16,681 patients with systolic heart failure or congestive heart failure (CHF) from twelve medical facilities in China [5]. The patients were split into groups with (n=6807) and without AF (n=9874). The research used different types of analyses to assess the value of SUA levels for AF, showing that higher SUA levels were linked to AF. Including levels in the prediction models improved their accuracy. The study concluded that SUA levels can help predict AF in CHF patients. Various studies consistently demonstrated that high SUA levels are connected to a risk of developing AF in HF patients. This connection is due to the role of SUA as an indicator of stress and inflammation, both of which contribute to the development of AF and HF. Elevated SUA levels might lead to dysfunction and atrial remodeling, increasing vulnerability to AF. Additionally, the use of diuretic furosemide, commonly used in managing HF, can worsen hyperuricemia by increasing acid reabsorption and reducing its excretion, thereby raising the risk of AF.

In the study by Birdal *et al.* (2023), which involved 538 heart failure patients, subjects were divided into two groups: those newly diagnosed with AF (n=133) and those without it (n=405) [6]. The investigation explored how SUA levels relate to the onset of AF. The findings indicated that patients with AF displayed elevated SUA levels (9.29 ± 2.93 mg/dL) compared to those without AF (7.67 ± 2.5 mg/dL). Moreover, there was a use of furosemide among individuals with AF. The comprehensive analysis suggested that both SUA levels and furosemide usage play a significant role in the development of AF, implying that hyperuricemia and diuretic administration are factors contributing to AF pathogenesis in heart failure patients (Table **1**).

These studies stand out for their design and large sample sizes. The study by Zhao *et al.* provided substantial statistical power to the results. Furthermore, rigorous inclusion and exclusion criteria were applied across the studies to ensure a defined patient group. The utilization of multivariate analyses aids in accounting for influencing factors, thereby enhancing the credibility of the conclusion.

However, there are certain constraints to consider. The retrospective nature of these studies introduces bias, like selection bias and information bias, which could impact the applicability of the results. Moreover, relying on records for data collection may also result in inaccurate information. Inflammatory markers, like CRP, which may contribute to both elevated SUA and incidence of AF, were not measured or compared between groups. The analysis also did not account for the use of medications other than diuretics that could influence SUA levels. Finally, although logistic regression identified independent associations, the use of Mendelian randomization in future studies could better determine whether SUA is a causal risk factor for AF by minimizing confounding and reverse causality. If causality is proven, comprehensive validation from longitudinal studies will be required to explore how medications like allopurinol that help lower SUA could potentially help enhance heart failure outcomes in patients with concurrent hyperuricemia and AF. Moreover, elevated SUA levels could serve as a biomarker for the risk of developing AF in patients with underlying HF, guiding early preventative strategies. Future prospective studies should incorporate inflammatory biomarkers, detailed medication use, and genetic analysis to more robustly assess the relationship between SUA and AF in patients with ischemic heart failure.

## Figures and Tables

**Table 1:** Patient population, demographics, comorbidities, and association of serum uric acid levels with atrial fibrillation in patients with heart failure.

**Author (Year)**	**Country**	**Population**	**Cohorts**	**Coh- orts Size**	**Age (Years)**	**Gender (n (%))**	**Comorbidities**	**LVEF%**	**Serum Uric Acid (SUA)**	**Diuretics Use**	**Association of SUA with AF in CHF Patients**		**Key Findings**	**Conclusion**	**Limitations**
											**Multivariate Analysis**	**Adjusted** **Variables**			
Tekin *et al.* (2012) [4]	Turkey	HF patients with and without AF	Patient with AF	78	71 ± 8	F: 30 (38%) M: 48 (62%)	HTN (71%) DM (20%) HLD (61%) Smoking (53%)	33.3 ± 6.6	6.4 ± 2.09	42 (56%)	OR 1.27 95% CI 1.06 ± 1.52 *p* = 0.01	Age Gender -Comorbidities: HTN, HLD, DM, smoking -Glucose, urea, creatinine, total cholesterol, triglycerides, SUA -Echocardiographic parameters: LVEF, LAD	Higher SUA levels in AF patients (6.4 ± 2.09 mg/dL) compared to non-AF (5.7 ± 1.9 mg/dL). SUA, age, left atrial diameter, and LVEDV were independently associated with AF.	SUA is an independent risk factor for AF in HF patients.	-Small sample size -Single-center retrospective study
			Patient without AF	285	66 ± 11	F: 137 (48%) M: 148 (52%)	HTN (75%) DM (28%) HLD (69%) Smoking (48%)	35.6 ± 5.4	5.7 ± 1.9	136 (48%)					
Birdal *et al.* (2023) [6]	Turkey	HF patients with and without AF	Patient with AF	133	Median 68 (62 - 72.5) Mean 68 ± 7.8	F: 51 (38.3%) M: 82 (61.7%)	HTN (24.8%) DM (8.2%) CAD (63.2%)	39.4 ± 11.9	9.29 ± 2.93	Thiazides 53 (39.8%) Furosemide 123 (92.5%) MRA 68 (51.1%)	OR 1.21 95% CI (1.09-1.35) *p* < 0.01	Age -Comorbidities: HTN, DM, CAD SUA -BB, furosemide, MRA, statin	SUA levels were significantly higher in AF patients (9.29 ± 2.93 mg/dL) than in non-AF patients (7.67 ± 2.5 mg/dL). SUA and furosemide use were associated with AF development.	Hyperuricemia may increase AF development in HF patients.	-Small sample size -Single-center retrospective study
			Patient without AF	405	Median 66 (58 - 74) Mean 66 ± 11.94	F: 152 (37.5%) M: 253 (62.5%)	HTN (25.9%) DM (9.4%) CAD (59%)	38.6 ± 13.9	7.67 ± 2.5	Thiazide 135 (33.3%) Furosemide 294 (72.6%) MRA 151 (37.3%)					
Zhao *et al.* (2012)	China	HF patients with and without AF	Patient with AF	6807	64.54 ± 13.61	F: 3324 (48.8%) M: 3483 (51.2%)	HTN (41.8 %) DM (23.7%) NYHA class III-IV (81.5%)	37.4 ± 12.7	2.58 ± 0.24	5071 (74.50%)	OR 1.549 95% CI 1.017 - 1.144 *p* < 0.01	Age Gender -Comorbidities: DM, NYHA class SUA -Echocardiographic parameters: LVEF, LAD, LVDD, RVDD ACEI, ARB, diuretics	Higher SUA levels were associated with AF. SUA levels significantly predicted AF with a sensitivity of 58.8% and specificity of 83.8%.	Elevated SUA levels are strongly associated with AF in HF patients and have been shown to improve the predictive accuracy of AF prediction models.	-Retrospective observational study
			Patient without AF	9874	62.19 ± 15.07	F: 3861 (39.1%) M: 6013 (60.9%)	HTN (51.6%) DM (11%) NYHA class III-IV (72.1%)	38.4 ± 13.9 6	2.56 ± 0.23	6123 (62.01%)					

**Abbreviations:** HF: Heart Failure, AF: Atrial Fibrillation, HTN: Hypertension, DM: Diabetes Mellitus, HLD: Hyperlipidemia, CAD: Coronary Artery Disease, NYHA: New York Heart Association, SUA: Serum Uric Acid, MRA: Mineralo- corticoid Receptor Antagonist, OR: Odds Ratio, CI: Confidence Interval, LAD: Left Atrial Diameter, LVEDV: Left ventricular End-Diastolic Volume, BB: Beta-Blocker, LVDD: Left Ventricular End-Diastolic Diameter, RVDD: Left Ventricular End-Diastolic Diameter, ACEI: Angiotensin-Converting Enzyme Inhibitor, ARB: Angiotensin II Receptor Blocker.

## LIST OF ABBREVIATIONS

AF: Atrial Fibrillation
CHF: Congestive Heart Failure
HF: Heart Failure
LA: Left Atrial
LV: Left Ventricular
SUA: Serum Uric Acid Level
